# Characterisation of Geogrid and Waste Tyres as Reinforcement Materials in Railway Track Beds

**DOI:** 10.3390/ma14154162

**Published:** 2021-07-27

**Authors:** Lihua Li, Yanan Fang, Bowen Cheng, Na Chen, Mi Tian, Yiming Liu

**Affiliations:** School of Civil Engineering, Architecture and Environment, Hubei University of Technology, Wuhan 430068, China; lilihua466@163.com (L.L.); zhumengcivil@163.com (Y.F.); chengbowen0926@163.com (B.C.); mitian0525@gmail.com (M.T.); ymliu@hbut.edu.cn (Y.L.)

**Keywords:** railway track bed reinforcement, triaxial test, stress–strain, shear strength, reinforcement strength ratio

## Abstract

The engineering behaviour of ballast is an important factor to determine the stability and safety of railway tracks. This paper examines the stress–strain, shear strength, peak deflection stress and reinforcement strength ratio of different reinforcement materials and reinforcement locations in ballast track bed layers based on large scale static triaxial shear tests. The results show that geogrid and waste tyre reinforcement have a significant effect on the peak deviator stress of railway track bed layers and the stress–strain relationship is strain-hardened. The peak deviator stress and shear strength of geogrid reinforcement are greater under the same conditions compared with waste tyres. The reinforcement of geogrid and waste tires increases the shear strength of the track bed significantly. The more layers of geogrid reinforcement, the more energy is required for the deformation of the track bed. The energy required for deformation is greater in the centre of the waste tyre than in the other reinforced forms, and the energy required for deformation is minimal in the fully reinforced form. Excessive tyre reinforcement changes the stiffness of the track bed layer, leading to an increase in the settlement rate. The reinforcement strength ratio between geogrid and waste tyre increases significantly with the increasing of the confining pressure and reinforcement layers. Moreover, the reinforcement strength ratio of the geogrid is significantly higher than that of the waste tyre.

## 1. Introduction

**Figure 1:**
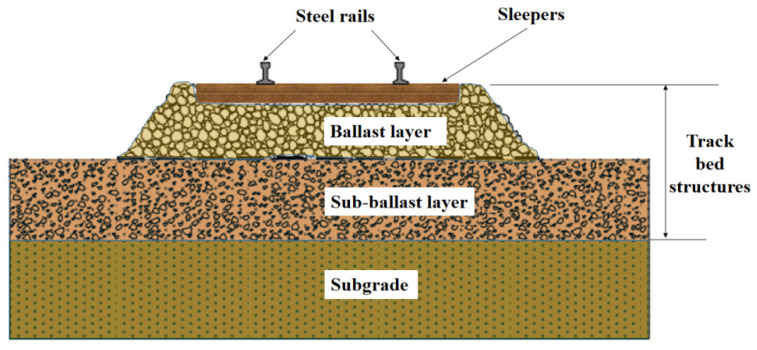
Ballast track structure.

The ballast railway track bed structure is composed of a ballast layer and a sub-ballast layer, which is filled with ballast particles of different grain sizes. In addition to its drainage function, ballast is an important substructure component of the track, providing lateral support and load transfer for the railway track, as shown in Figure 1. The track bed layer is based primarily on the interlocking of ballast aggregate particles to distribute the wheel loads to the top of the track bed, or to the subgrade beneath it, to an acceptable level. Excessive load of the running train will cause the vertical and lateral displacement of the railway track bed, which will eventually lead to excessive settlement and instability of the track bed. The railway department must take effective measures to solve the above problems, which may cause a series of safety problems such as train derailment and track misalignment.

Methods of controlling roadbed deformation and settlement by reinforcement in railway or road engineering are gaining increasing attention [1,2,3,4,5]. Geogrids are commonly used in roads and railways to isolate, reinforce and reduce uneven settlement, and limit deformation [6,7]. The shear strength and modulus properties of the ballast layer can be improved and the lateral movement of ballast particles can be limited by geogrid reinforcement in ballast layers. The geogrids in the track bed also perform a stabilization function, and vertical settlement and lateral displacement are also reduced by effective interlocking of the geogrid and the ballast [8,9]. The use of a waste tyres in the capping layer provides considerable lateral confinement to the infilled materials, thus contributing to a reduction in lateral spreading and vertical settlement of particles [10,11]. In addition, using waste tires as reinforcement material can make effective use of solid waste material, reduce environmental pollution and effectively solve the problem of its disposal in piles.

Geogrids and waste tyres are used as reinforcing material in the railway track bed layer in many studies. They can decrease the settlement and deformation of the railway track bed, thus reducing railway track maintenance costs during railway operation [12,13,14,15,16,17]. Some scholars have carried out experimental research on the track bed structure reinforced by geogrid: Sadeghi et al. [18] conducted a large straight shear test showing that geogrid reinforcement significantly increased the shear strength and vertical stiffness of the contaminated ballast layer. Hussaini et al. [19] showed that the geogrid reinforcement prevented the lateral displacement of ballast particles and reduced the vertical settlement of the ballast layer through cyclic tests. Brown et al. [20] described a series of tests of geogrid reinforcement of railway ballast, which increased the shear strength of geogrid-reinforced ballast by 50% and the stiffness by 2.5%. Woodward et al. [21], using polymer-reinforced cyclic tests, showed that the load increase for a given polymer-reinforced ballast particle may be about 40%. Kennedy et al. [22] obtained results from experimental tests showing that the 3D geocell reinforced ballast layer significantly reduced the settlement of the track structure.

In addition, some scholars have conducted research on the structure of the track bed reinforced with waste tires: Gong et al. [23] conducted a straight shear test that used tire chips with a mixture of railroad ballast filler, and the results showed that tire chip-reinforced ballast reduced the breakage of ballast particles. Esmaeili et al. [24] added tire spacers under sleeper pads, resulting in reduced track settlement and ballast particle fragmentation index. Mohajerani et al. [25] performed seismic simulations in which rubber–soil mixtures were used as the foundation and structural interface, which reduced ground acceleration by 60–70% in both vertical and horizontal directions. Indraratna et al. [26] studied rubber tire-reinforced ballast layers in triaxial tests, and the results showed that rubber tire reinforced ballast layers can reduce ballast degradation and the particle movement of the track substructure. Li et al. [27] studied waste tire-reinforced construction waste soil, and the test proved that the lateral binding force provided by the waste tire can enhance the mutual occlusion of soil and reduce the accumulated strain in the axial direction. Akbulut et al. [28] noted that the addition of rubber tire fragments to clay soil resulted in an increase in the shear modulus and cohesion of the reinforced soil mass. Rao et al. [29] conducted triaxial tests by changing the size and content of tire fragments. The results demonstrated that sand-waste tyres mixtures of up to 20% concentration could be a potential material for highway construction and embankment construction projects reaching up to around 10 m in height.

Although there are a lot of research studies on geogrids and waste tyres, they both have limitations. Most of the previous studies had a number of shortcomings: (1) reinforcement tests were only carried out on the ballast layer or the sub-ballast layer alone; (2) some tests that simulate the actual track structure layer or field tests usually test the effect of the reinforcing material at the ballast and sub-ballast interface; (3) the effect of changes in the location and amount of geogrid and waste tyre reinforcement on the entire track bed has not been tested. Therefore, it is necessary to establish a large-scale triaxial test that simulates the actual railway track bed structure, including a comprehensive effect and mechanism of action on the different reinforcement materials and different reinforcement positions of the track bed. This paper takes geogrid and waste tyre reinforced track beds as the objects of study based on a large static triaxial test. The tests were carried out by different perimeter pressures, reinforcement materials, reinforcement forms and reinforcement material locations. Geogrids and waste tires were selected as ballast reinforcement materials in the experiment, and the reinforcement effects were compared and analyzed. Through reinforcement at different positions of the track bed, the effects of the reinforcement positions of the two materials and the amounts of reinforcement on the strength and deformation characteristics of the track bed are discussed.

## 2. Test Equipment and Materials

### 2.1. Test Equipment

**Figure 2:**
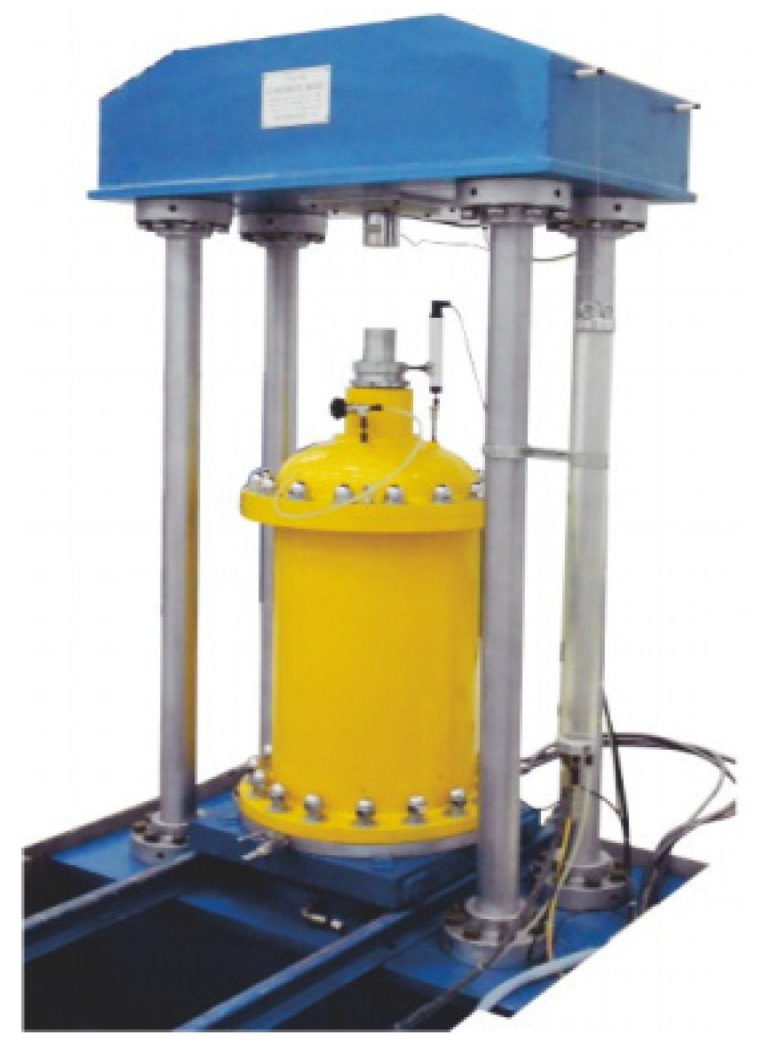
SZ30-4DAD large static triaxial test equipment.

In this study, the SZ30-4DAD large static triaxial test equipment for coarse-grained soils was used, as shown in Figure 2. The equipment consists of computer host, static axial pressure stabilization system, pressure chamber, confining pressure stabilization control system, and automatic data acquisition system. The high-precision and low-speed CNC metering oil source speed regulation system was used to complete the axial stress and strain loading. The maximum axial static load was 21 MPa, the maximum confining pressure was 4.0 MPa, and the sample size was 300 × 600 mm.

### 2.2. Test Filler

**Figure 3:**
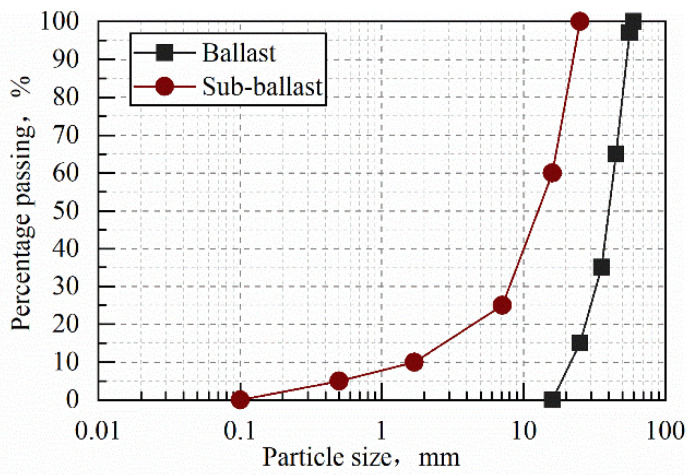
Test ballast and sub-ballast grain size gradation curves.

The test railway ballast was taken from the graded gravel of the track bed at the track bed quarry site, and the parent rock was granite. The materials of the ballast layer and the sub-ballast layer were screened and prepared according to the “Code for Design of Heavy-Duty Railways” (TB10625-2017) [30], and the real track bed particle gradation curve was simulated, as shown in Figure 3. Through the heavy compaction test, the basic physical parameters of the maximum dry density and optimal moisture content of the ballast and sub-ballast materials are shown in Table 1.

### 2.3. Reinforcement Materials

**Figure 4:**
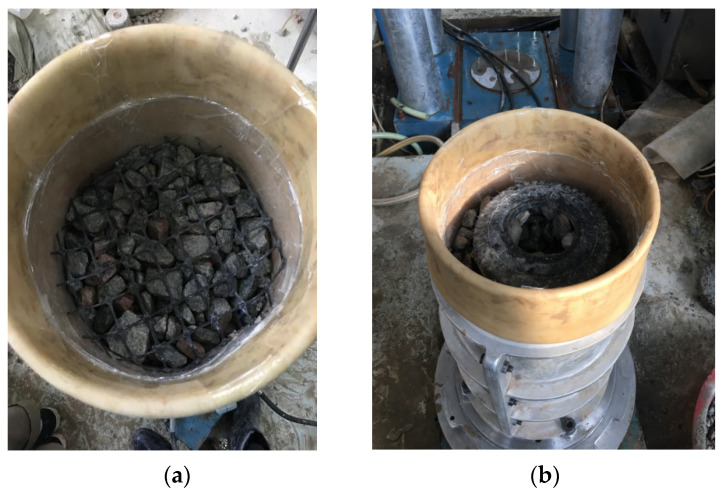
Test reinforcement material: (**a**) geogrid; (**b**) tyres.

In this paper, TGSG-3030 type geogrid and small electric vehicle tires are used as large triaxial test reinforcement materials, respectively. Geogrid and tire were placed horizontally in the sample, and the layout of the reinforcement is shown in Figure 4. The physical and mechanical indexes of the geogrid and waste tire are shown in Table 2 and Table 3.

## 3. Test Procedure and Program

### 3.1. Sample Preparation

**Figure 5:**
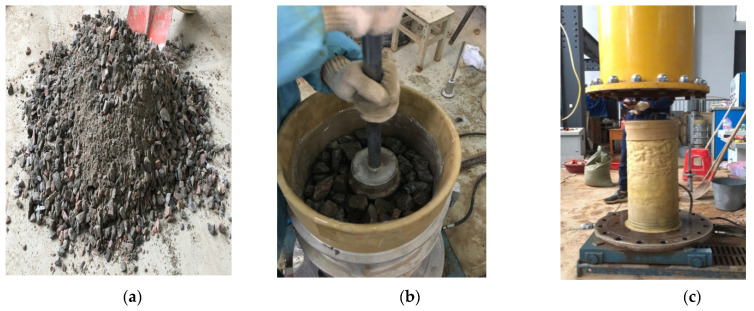
Sample preparation process: (**a**) mixing; (**b**) compaction of the sample; (**c**) vacuum hood pressure chamber after evacuation.

**Figure 6:**
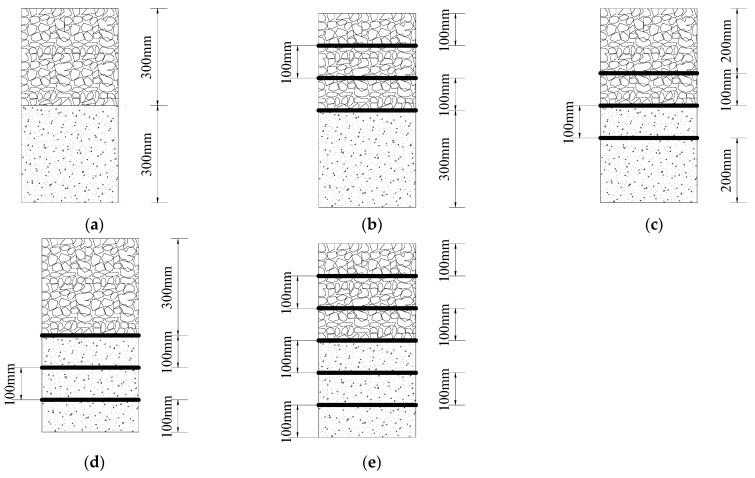
Geogrid and waste tyre reinforcement arrangement: (**a**) unreinforcement (UR); (**b**) upper reinforcement (BGT1/T1); (**c**) central reinforcement (BGT2/T2); (**d**) lower reinforcement (BGT3/T3); (**e**) all reinforced (BGT5/T5).

The diameter of the sample was 300 mm and the height was 600 mm. The diameter of the sample was six times the maximum particle size of the graded gravel, and this experiment was not affected by size effects. Before the test, the fillers were mixed and smothered. The treated fills were compacted in six layers. After compaction of the samples, the samples were kept upright by means of vacuuming. The test preparation process is shown in Figure 5. When the negative pore water pressure reached 70–80 kPa, the sample was stopped from vacuuming, and the pressure chamber was installed and flushed into the pressure chamber. Then, the samples were relieved of pore water pressure and a 40 kPa confining pressure was applied to avoid collapse of the samples. The sample saturation was carried out in two ways: vacuum saturation and head saturation. By applying confining pressure, the ratio of confining pressure increment to pore pressure increment was calculated, the pore pressure coefficient B value was measured, and the saturation was controlled above 95%. The samples were subjected to isotropic consolidation by applying confining pressures of 50, 100 and 150 kPa, and the consolidation could be stopped and the test could be started when the consolidation drainage was stable. Reinforced material geogrid and tires were placed horizontally in the samples; the arrangement of reinforcement is shown in Figure 6. In order to simulate the actual work condition of the graded gravel fill in track bed layer, the test was carried out with a moisture content close to the optimal 5%. The samples were prepared according to the compaction requirements of the graded gravel fill in the actual heavy-duty railway track bed, and the compaction coefficient was taken as 0.95. The sample preparation process was carried out with reference to the Geotechnical Test Procedure (GB/T 50123-2019) [31] and the Geotechnical Test Procedure for Railway Engineering (TB10102-2010) [32]. 

### 3.2. Test Program

This test simulates the real railroad track bed conditions and adopts the consolidation without drainage (CU) static triaxial test. Referring to the Standard for Geotechnical Test Methods, the test is stopped when the track bed samples collapse during the shear process or when the accumulated strain in the axial direction reached 15% of their initial height. The geogrid and waste tyre reinforcement track bed were arranged in the form of upper (BGT1/T1), central (BGT2/T2), lower (BGT3/T3) and all (BGT5/T5). Reinforced material geogrid and tires were placed horizontally in the samples, and the arrangement of reinforcement was shown in Figure 6. The specific test program is shown in Table 4.

## 4. Test Results and Analysis

### 4.1. Shear Damage Characteristics of Samples at Different Reinforcement Positions

**Figure 7:**
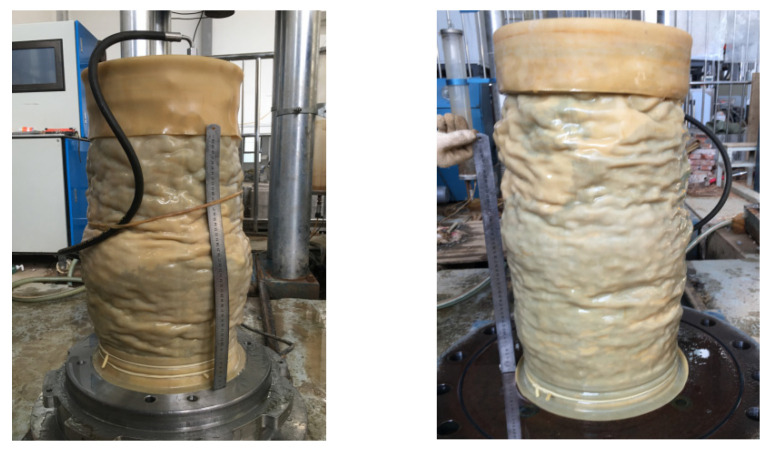

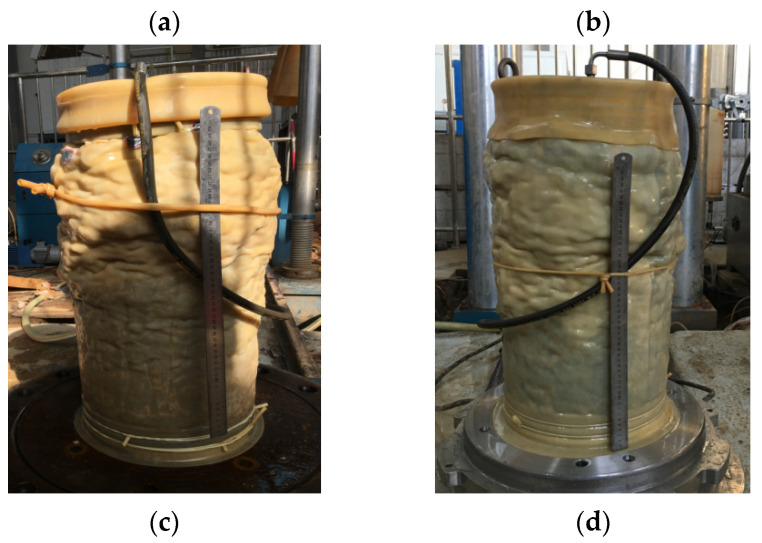
Shear damage characteristics of specimens at different reinforcement locations: (**a**) BGT/T1; (**b**) BGT/T2; (**c**) BGT/T3; (**d**) BGTG/T5.

Figure 7 shows part the damaged state of the samples after reinforcement at different positions. The geogrid and waste tires were used as reinforcement in the samples composed of a track bed layer mixture at different positions and under different confining pressures, and the SZ30-4DAD automatic triaxial instrument was used for the shear test of coarse-grained soil. It can be seen from Figure 7 that the shear failure of the track bed sample does not reflect the shear surface theoretically, and it can be clearly observed that the sample exhibits dilatancy failure. The cross-sectional diameter of the track bed sample increases and forms a clear “packet” pattern. Comparing and analyzing the samples of the track bed, it can be seen that the “packet” phenomenon is related to the different reinforcement positions of the track bed. Under the action of shearing force, the diameter of the reinforced position of the track bed is significantly smaller than the diameter of the unreinforced position of the track bed. Under different confining pressures, the higher the confining pressure, the smaller the “packet” diameter of this shear swelling phenomenon. The reason for the dilatancy phenomenon of the track bed samples is that the ballast particles bite and squeeze each other under the load of the track. The positions of the ballast particles change and the pores between the particles become tighter. Reinforcement at different locations in the bed layer results in greater ballast particle occlusion and interlocking forces at the reinforced locations. The interlocking effect between the reinforcement material and the ballast particles inhibits the lateral displacement of the particles.

### 4.2. Stress–Strain Relationship Curve for Geogrid and Waste Tyre Reinforcement Track Bed Layer

**Figure 8:**
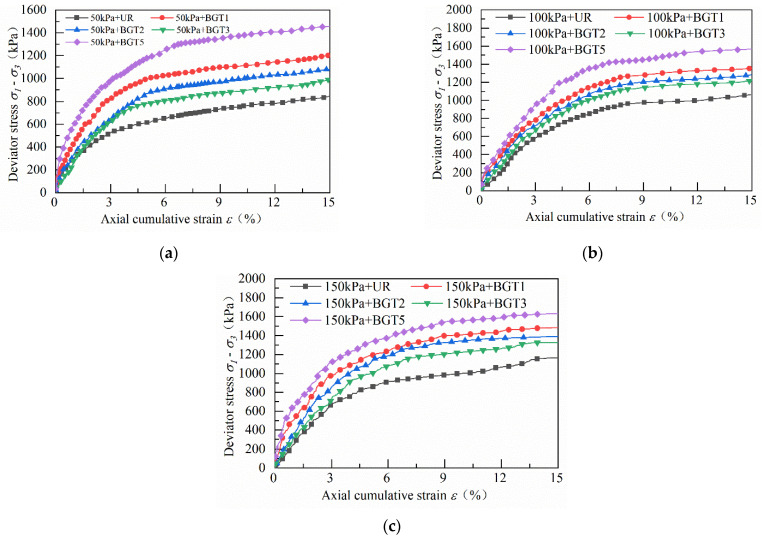
Stress–strain relationship curves for geogrid reinforcement track bed layers at three different confining pressures: (**a**) 50 kPa geogrid reinforcement; (**b**) 100 kPa geogrid reinforcement; (**c**) 150 kPa geogrid reinforcement.

Figure 8 shows the static triaxial test stress–strain curves for the track bed samples under 50 kPa, 100 kPa and 150 kPa confining pressure, respectively. The stress–strain curves do not show a peak maximum deviator stress, so the deviator stress at 15% of the axial accumulated strain is taken as the peak deviator stress. As can be seen from the graph, the track bed samples are subjected to shear stress and the peak deviator stress increases with the increase in axial cumulative strain. When the axial cumulative strain has reached about 3%, the increase rate of the deviator stress becomes slower, and the track bed layer shows strain hardening and shear expansion characteristics throughout the shear process. Figure 8 shows that as the geogrid reinforcement position changes, the peak deflection stress of the ballast specimen also changes. When the numbers of reinforced layers of the track bed layer samples were the same, it was found that the reinforcement effect of BGT1 was significantly higher than that of BGT2 and BGT3. At 50, 100 and 150 kPa, the BGT1-reinforced peak deviator stresses increased by 43.1%, 27.3% and 26.8%, respectively, compared to the unreinforced samples. The best reinforcement effect was observed when the BGT5 reinforced ballast samples were reinforced; the BGT5 peak deviator stresses increased by 73%, 47.3% and 40.2%, respectively, compared to the unreinforced samples under 50, 100 and 150 kPa. The test results show that the pores between the particle frameworks of the track bed are relatively large at the beginning of the static load, and the mutual displacement and squeezing of the ballast particles under the static load result in the rapid development of the axial cumulative strain of the ballast bed in the early stage. With the action of static loading, the interaction between particles produces a certain amount of particle wear and pressure density, and the broken fine ballast particles fill the pores between ballast particles. Thus, the contact surface between the ballast particles and the geogrid increases, raising the sliding friction and bite friction between the ballast particles. The geogrid reinforced ballast particles provide continuity, and the high tensile strength of the geogrid and its interlocking effect increase the shear strength of the ballast particles. The structure where the ballast particles and the geogrid intersect each other has a larger stress diffusion angle, which homogenizes the stress distribution of the entire track bed sample, thereby increasing the peak deviator stress of the track bed. The lateral restraining force of the geogrid on the ballast bed restricts the lateral deformation of the ballast particles. Meanwhile, the axial deformation resistance of the ballast is enhanced and the accumulated strain in the axial direction of the track bed is reduced. The confining pressure has a significant effect on the axial deformation of the ballast bulk. The axial strain in the ballast bulk increases as the confining pressure decreases. The lateral restraint effect of the geogrid unit on the tension of the ballast particles is similar to the lateral restraint effect provided by the confining pressure, which has positive significance for controlling the ballast deformation.

**Figure 9:**
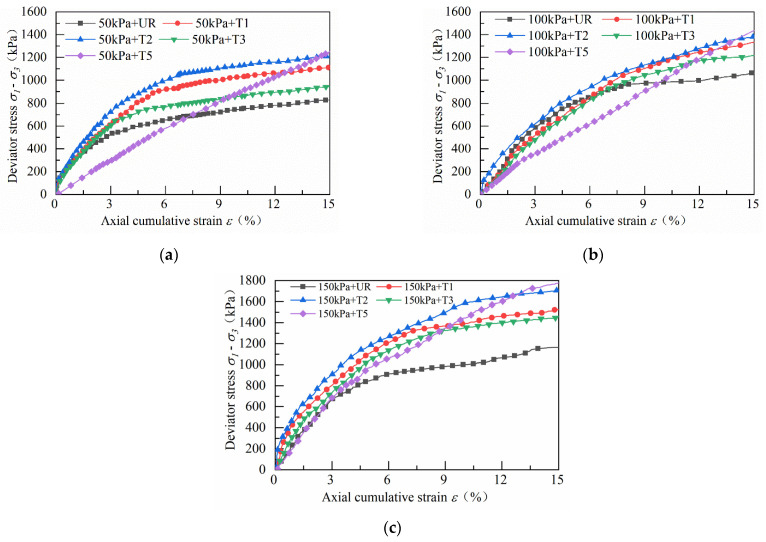
Stress–strain relationship curves for waste tyre reinforced track bed layers at three different confining pressures: (**a**) 50 kPa waste tyre reinforcement; (**b**) 100 kPa waste tyre reinforcement; (**c**) 150 kPa waste tyre reinforcement.

Figure 9 shows that the peak deviator stress increases with the increasing axial cumulative strain in the waste tyre reinforcement track bed layer under shear stress at three different confinement pressures. In the early stages, the axial cumulative strain increases rapidly with the increase in deviator stress. When the axial cumulative strain reached about 6%, the increase rate of the deviator stress became slower. The track bed layers of the reinforced forms T1, T2, T3 and T5 consistently exhibited strain hardening and shear expansion. When the same reinforcement conditions were applied, the reinforcement of T2 was significantly more effective than that of T1 and T3. The peak deviator stresses for T2 increased by 44%, 29.9% and 42% under different confining pressures compared to the unreinforced track bed. Under T5 reinforcement conditions, the deviator stress of the track bed increased linearly under the confining pressures of 50 kPa and 100 kPa. As rubber replaces the original granite ballast, the stiffness of the ballast bed specimens decreases, and the anti-deformation ability of the ballast bed decreases during the shearing process. The peak deviator stress of the waste tyre reinforcement track bed layer increases as the confining pressure continues to increase. Higher confining pressures help to reduce the lateral and axial strains in the track bed samples. The restraint provided by the tyre unit will help to increase the stiffness of the enclosed ballast, which helps to reduce the axial accumulation of strain in the track bed. In the same way, the additional lateral restraint provided by the tyre cell will prevent lateral movement of the ballast material, which helps to reduce the deformation of the track bed.

A comparative analysis between Figure 8 and Figure 9 shows that under the confining pressure conditions of 50, 100 and 150 kPa, the peak deviator stresses increase by 43.1%, 27.3% and 26.8% for BGT1 reinforced compared to unreinforced samples, 27.9%, 20.6% and 19.2% for BGT2 reinforced, and 18.4%, 14.6% and 13.9% for BGT3-reinforced. The peak stresses in BGT5 increased by 73%, 47.3% and 40.2%, respectively. Compared with the unreinforced samples, the T1 peak deviator stresses of the used waste tyre reinforced track bed samples increased by 31.2%, 25.6% and 30.1%, the T2 peak deviator stress increased by 44%, 29.9% and 42%, the T3 peak deviator stress increased by 24.2%, 14.6%, and 20.9%, and the T5 peak deviator stress increased by 48.6%, 35.5%, and 47.8%, respectively. From the growth of the deviator stress of the two material reinforced track bed samples, it can be concluded that the geogrid reinforcement effect is more significant than that of waste tires under low confining pressure. The reinforcement effect of waste tires is better under high confining pressure.

### 4.3. Shear Strength Parameters for Geogrid and Waste Tyre Reinforced Track Bed

**Figure 10:**
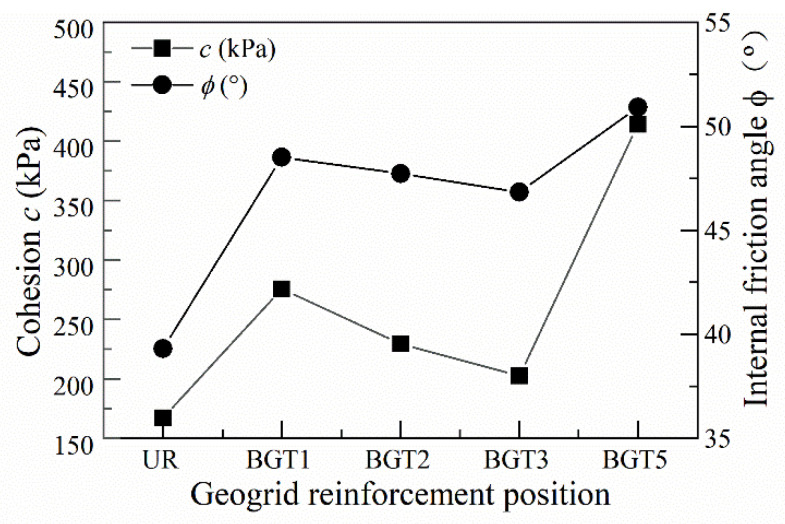
Effect of geogrid reinforcement position on shear strength.

Figure 10 shows that different geogrid reinforcement positions have a significant impact on the anti-shear strength parameters. It can be concluded that when the geogrid reinforcement was compared with the unreinforced track bed, the cohesive force and internal friction angle are both increased. When geogrid BGT1 was reinforced, the angle of internal friction was greater than that of BGT2 and BGT3 and the cohesion was relatively higher. When the geogrid was BGT5 reinforced, the angle of internal friction and cohesion were greatest. As a result of the interlocking and interlocking of the ballast particles and the geogrid, the ballast particles are broken and fractured to fill the pores between the particles. Because of the tensile force of the geogrid acting on the ballast particles in contact with it, the cohesion between them and the angle of internal friction increases. The internal friction angle has a greater influence on the shear strength of coarse ballast in the geogrid reinforced track bed, while the cohesion has a relatively small influence on the shear strength of the track bed. The reinforcement effect was best when geogrids BGT1 and BGT5 were reinforced in the track bed, with maximum cohesion and internal friction angle. Geogrid reinforcement can also improve the shear strength of the track bed layer, and the comparative effect of geogrid reinforcement is BGT5 > BGT1 > BGT2 > BGT3.

**Figure 11:**
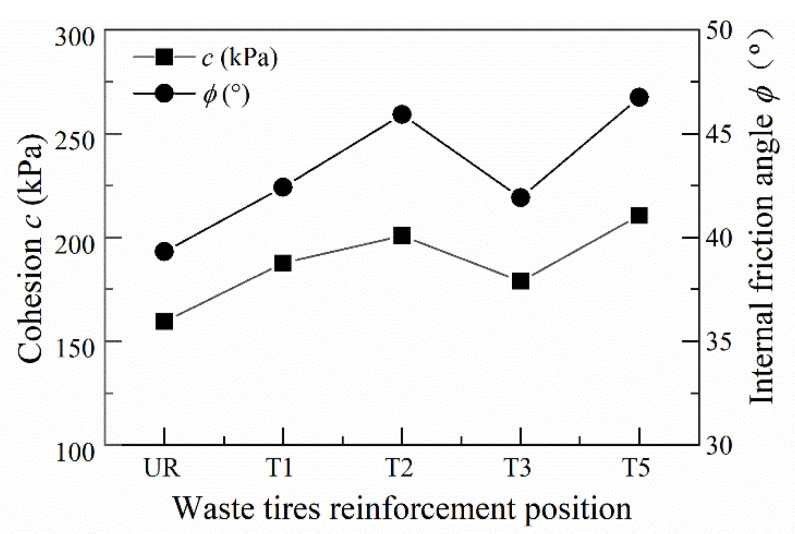
Effect of waste tyre reinforcement position on shear strength.

Figure 11 shows the significant influence of the different reinforcement positions on the shear strength parameters of the waste tyre. The cohesive force and the internal friction angle both tend to increase when the waste tyre is reinforced compared to the unreinforced samples. T1 has a higher internal friction angle and greater cohesion than T2 and T3, indicating that the shear strength of the reinforced tyre is better at the interface of the track bed than other locations. The cohesion and the internal friction angle are both at their maximum for T5 reinforcement. Because the contact surface between the waste tyre and the ballast particles is the largest in T5, the ballast particles and the surface and inner side of the waste tyre are mutually constrained and interlocked, resulting in an increase in the internal friction angle and cohesion of the track bed. Compared to other forms of waste tyre reinforcement, T2 has a better reinforcement effect and a significant advantage in terms of shear strength. Considering that waste tyre is a good three-dimensional reinforcing material, it can provide sidewall binding and frictional forces that increase the particle-to-particle bite, which increases the ballast bulk particle cohesion and friction angle. When ballast particles are broken, tyre reinforcement can limit the lateral and vertical development of cracks and improve the integrity and continuity of the track bed.

### 4.4. Energy Absorption in Geogrid and Waste Tyre Reinforced Track Beds

Energy absorption was the amount of energy required to cause deformation in geogrid and waste tyre reinforced track beds [33]. This paper used the energy absorption required for deformation to quantify and compare the effect of the location of geogrid and waste tyre reinforcement on the deformation capacity of the track bed. This has been determined by calculating the area under the stress curve. In this study, energy absorption was calculated for 10% of the axial strain in all tests.

**Figure 12:**
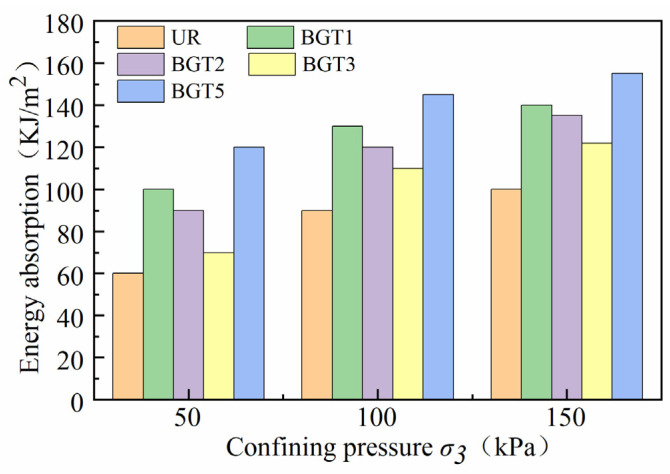
Energy absorption of geogrid reinforced track bed.

The effect of deformation of the geogrid reinforced track bed layers on energy absorption under different confining pressures and locations is shown in Figure 12. Under 50 kPa, 100 kPa and 150 kPa confining pressure constraints, BGT1 has significantly greater energy absorption compared to BGT2, BGT3 and unreinforced track bed. It can be concluded that geogrids have different energy absorption capacities at different reinforcement locations in the track bed, and that geogrids can improve the energy absorption of the track bed and thus increase the deformation resistance. The energy absorption of ballast was much higher with the BGT5 reinforced method than with the other reinforced methods. Compared to unreinforced ballast, the energy absorption is 71%, 61% and 55% higher, respectively. The difference in energy absorption at the geogrid reinforced locations increases as the confining pressure increases. This indicates the interaction between the geogrid reinforcement position and the confining pressure on energy absorption. The effect of the geogrid reinforcement position on energy absorption increases with increasing confining pressure. Under higher confinement pressures, more energy is required to deform the roadbed with more layers of geogrid reinforcement. Under low confining pressure, geogrid reinforced track bed has better anti-deformation ability and absorption effect capacity than it does under high confining pressure.

**Figure 13:**
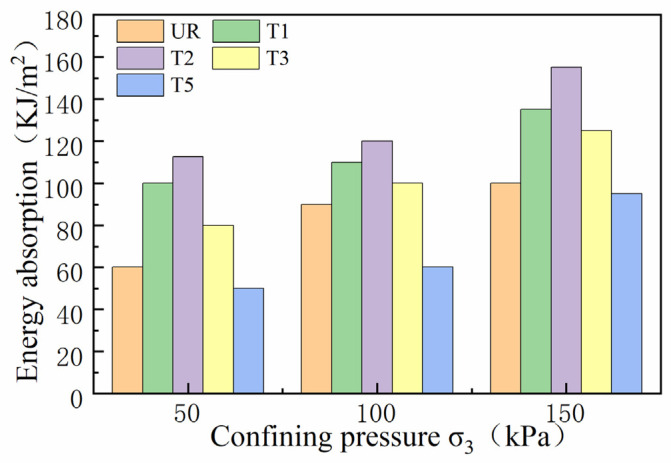
Energy absorption of waste tire reinforced track bed.

The effect of different confining pressures and different locations of the waste tyre reinforced track bed layer on energy absorption is shown in Figure 13. The waste tyre reinforcement was significantly different from the geogrid, with the smallest energy absorption across the ballast when T5 reinforcement was applied. Because the waste tyres were composed of flexible rubber material, the maximum number of T5-reinforced tyres resulted in a smaller stiffness of the track bed samples, so the minimum amount of energy was required for the deformation of the track bed. The energy absorption of T2 reinforcement was better than that of T1 and T3 when compared to T2 and T3 under 50 kPa, 100 kPa and 150 kPa circumferential pressure. The deformation required to absorb energy was increased by 70.8%, 40% and 55% for T2. The T2 tyre provides additional restraint and frictional resistance; in addition, the T2 tyre reinforcement leads to an increase in the stiffness of the bed layer and the bed deformation under static load requires the greatest energy absorption. As the confining pressure increases, the reinforcement of the waste tyre increases the energy absorption and the resistance to deformation of the track bed.

### 4.5. Reinforcement Strength Ratio under Different Confining Pressures

To assess the effect of different confining pressures, reinforcement materials and reinforcement locations on the reinforcement strength of the track bed, the reinforcement material strength ratios (*RSR*) were defined as follows (1).

(1) $$RSR=\frac{{\left(\sigma_{1}-\sigma_{3}\right)}^{R}}{{\left(\sigma_{1}-\sigma_{3}\right)}^{UR}}$$

where (*σ*_1_ − *σ*_3_)*^R^* is the peak deviator stress in the geogrid and waste tyre reinforced track bed, (*σ*_1_ − *σ*_3_)*^UR^* is the peak deviator stress in the unreinforced track bed.

**Figure 14:**
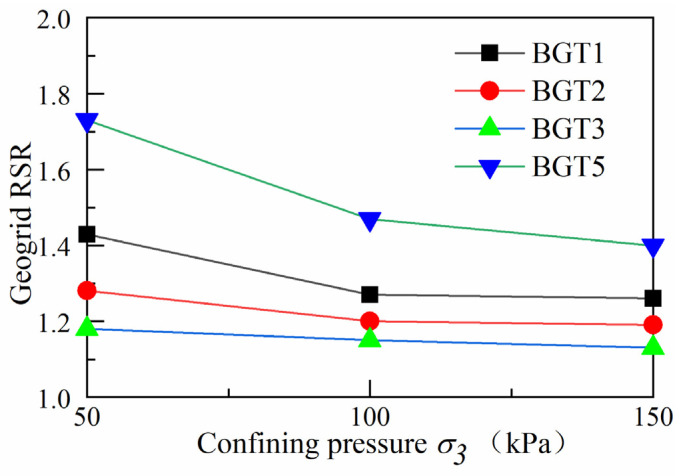
Geogrid reinforcement strength ratio.

According to the reinforcement strength parameters, the reinforcement strength ratio of the track bed can be obtained under different water levels of confining pressure, as shown in Figure 14. The reinforcement strength ratio of the geogrid decreases significantly as the confining pressure increases, indicating that the geogrid reinforced track bed has the best reinforcement effect at low confining pressure. The reinforcement strength ratio of BGT1 increased by 43%, 27% and 26%, the reinforcement strength ratio of BGT2 increased by 28%, 20% and 19%, the reinforcement strength ratio of BGT3 increased by 18%, 15% and 13%, the reinforcement strength ratio of geogrid varied with the location of reinforcement, and the reinforcement strength ratio of BGT1 had the best effect. The reinforcement strength ratio of BGT5 increased by 73%, 47% and 40%, respectively. The increased amount of reinforcement in the geogrid leads to a significant increase in the reinforcement strength of the track bed layer. For example, BGT5 at 50 kPa confining pressure increases significantly, by 73%, compared to unreinforced track bed. The shear restraint effect of the gripping and sliding friction between the geogrid and the ballast particles decreases as the confining pressure increases, so the reinforced strength ratio of the geogrid decreases as the confining pressure increases.

**Figure 15:**
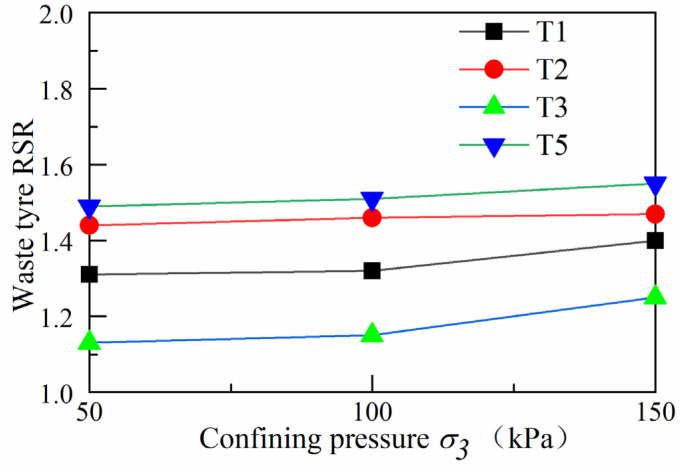
Waste tyre reinforcement strength ratio.

Figure 15 shows that the reinforcement strength ratio of the waste tyre increases significantly with the increasing circumferential pressure. The reinforcement strength ratio of T2 was greater than that of T1 and T3 at 50, 100 and 150 kPa confining pressures, T2 was more effective at different locations, and the reinforcement strength ratio of T2 was increased by 44%, 46% and 47% compared to the unreinforced track bed. As the number of reinforced waste tyres increases, the reinforcement strength ratio of the waste tyres increases significantly. The reinforcement strength of T5 increases by 49%, 51% and 55%. This was because the scrap tyres are a rubber material with less strength and stiffness than granite ballast. The overall stiffness of the track bed was reduced due to the replacement of a certain volume of ballast material during the reinforcement of the waste tyres. With the increase in perimeter pressure, the ballast and tyre friction and squeezing were increased, leading to an increase in the stiffness of the track bed and an increase in the track bed deformation strength.

## 5. Conclusions

In this work, the stress–strain and shear strength characteristics of the reinforced railway track bed were investigated through large static triaxial tests. This paper takes geogrid and waste tyre reinforced railway track bed as the research topic, and discusses the deformation characteristics of railway track bed under different confining pressures, with different reinforced materials and reinforced positions. A reinforcement method to reduce the settlement of the railway track bed is also provided. The main research results are as follows:

(1) When the geogrid was reinforced at different locations in the track bed, the peak deviator stress in the track bed increased as the axial strain gradually increased. The whole change process was in a strain-hardening state. BGT1 has a higher peak deviator stress than the BGT2 and BGT3 reinforcement methods, and BGT1 has a better reinforcement effect than BGT2 and BGT3.
(2) When waste tyres were reinforced in different positions of the roadbed, the effect of waste tyre reinforcement was not obvious at lower axial cumulative strains. As the axial cumulative strain increased, the roadbed remained strain-hardened for T1, T2, T3 and T5 reinforcement forms. T2 reinforcement forms were significantly better than T1 and T3.
(3) The internal friction angle and cohesion of the geogrid and waste tyre reinforced track bed layers increased significantly. The shear strength of the BGT1 and T2 reinforced track bed layer was significantly better than the reinforcement at other locations. The best results in terms of shear strength were obtained when the reinforcement was in the form of BGT5 and T5.
(4) The resistance to deformation of geogrid and waste tyre reinforced track beds is related to energy absorption. The more energy absorption of geogrid and waste tyre reinforced track beds, the greater their resistance to deformation. BGT1 requires the most energy absorption for deformation than BGT2 and BGT3. More energy is required for deformation of roadbeds under the bgt5 reinforcement method. The energy absorption required for the deformation of the waste tyre under stress was greatest when the waste tyre was reinforced by the T2 reinforcement method.
(5) In the railway bed layer, geogrids and waste tyres have a significant reinforcing effect when placed, as reinforcing material, in the track bed layer.

## Figures and Tables

**Table 1:** Physical properties of ballast and sub-ballast.

Packing Classification	Max Dry Density/(g·cm^−3^)	Optimum Water Content/(%)	*C_u_*	*C_c_*
ballast	2.18	0.79	6.32	1.16
sub-ballast	1.87	4.8	9.41	2.6

**Table 2:** Engineering properties of biaxial geogrid.

Type	Ultimate Tensile Strength/(kN·mm^−1^)		Ultimate Elongation/%		Tensile Strength at Different Elongations in the Vertical/((kN·mm^−1^)		Tensile Strength at Different Elongations in the Horizontal/(kN·mm^−1^)	
	MD	CMD	MD	CMD	2%	5%	2%	5%
TGSG–3030	30	30	≤16	≤13	≥11	≥13	≥15	≥15

**Table 3:** Engineering properties of tires.

Vertical Tensile Yield/(kN·m^−1^)	Vertical Yield Elongation/%	Single Tyre Size D × W × H/mm × mm × mm	Tensile Modulus at Different Strains/(kN·m^−1^)	
			2%	5%
54.6	75.8	170 × 50 × 50	33.2	78.1

**Table 4:** Design program for static triaxial tests.

Samples No.	Confining Pressures *σ*_3_/kPa	Reinforced Materials	
		Geogrid	Waste Tyres
1	50	UR	UR
2		BGT1	T1
3		BGT2	T2
4		BGT3	T3
5		BGT5	T5
6	100	UR	UR
7		BGT1	T1
8		BGT2	T2
9		BGT3	T3
10		BGT5	T5
11	150	UR	UR
12		BGT1	T1
13		BGT2	T2
14		BGT3	T3
15		BGT5	T5
